# miR-543 Inhibits the Occurrence and Development of Intrauterine Adhesion by Inhibiting the Proliferation, Migration, and Invasion of Endometrial Cells

**DOI:** 10.1155/2021/5559102

**Published:** 2021-03-30

**Authors:** Xin Liu, Qian Xu, Chao Chen, Hua Duan

**Affiliations:** Beijing Obstetrics and Gynecology Hospital, Capital Medical University, Gynecological Mini-Invasive Center, 17 qihelou st, Dongcheng District, Beijing 100006, China

## Abstract

**Objective:**

To explore the function of miR-543 in endometrial cells and the possible mechanism of regulating the occurrence and development of intrauterine adhesion.

**Method:**

Endometrial epithelial cells and endometrial adenocarcinoma cells were transfected with miR-543 mimics and miR-543 inhibitor as the experimental group and were tested with the control group, using the CCK-8 method, scratch test, and Transwell assay, and flow cytometry was used to detect the proliferation, migration, invasion, and apoptosis of cells. RT-qPCR and Western blot were used to detect the expression of corresponding mRNA and protein.

**Results:**

After the overexpression of miR-543, endometrial epithelial cells and endometrial adenocarcinoma cells have reduced migratory, proliferative, and invasive capabilities, while the apoptosis rate has increased significantly. The mRNA expression of *CDH2*, *COL16A1*, *vimentin*, *α-SMA* and *fibronectin* decreased, and the protein expression of CDH2, vimentin, and *α*-SMA also decreased, while the mRNA and protein expression of CDH1 increased. The result after interfering with miR-543 is opposite, and luciferase reporter gene confirms that *CDH2* is the target gene of miR-543.

**Conclusion:**

During the formation of intrauterine adhesions, the expression of *CDH2*, *COL16A1*, *vimentin*, and *α-SMA* may be inhibited by the high expression of miR-543, which may affect the degree of fibrosis and collagen content in the intrauterine adhesions, thereby inhibiting the occurrence and development of intrauterine adhesions.

## 1. Introduction

Intrauterine adhesions (IUA), also known as intrauterine adhesion syndrome, is a disease caused by postpartum infections, low estrogen levels, uterine surgery, etc. in the endometrial basement membrane shedding and damage, resulting in partial or complete obstruction of the uterine cavity or cervical canal [1]. It is characterized by abnormal menstruation, infertility, recurrent abortion, and other pregnancy-related complications [2, 3]. The main pathogenic factors of IUA were induced abortion, incomplete abortion, curettage of uterus in missed abortion, postpartum hemorrhage, and genital tuberculosis [4]. IUA is a fibrotic disease caused by tissue injury. At present, moderate and severe uterine adhesions have a high recurrence rate and poor prognosis, which is one of the difficulties in treatment. It is necessary to further study its pathogenic mechanism.

Improvement of uterine adhesion can restore the normal shape of uterine cavity, promote the repair of endometrium, and finally improve the fertility of patients with uterine adhesion. Many genetic and environmental factors influence the formation and development of intrauterine adhesions. MicroRNA (miRNAs) is a short strand RNA discovered in recent years, which can negatively regulate the target gene and participate in all aspects of life activities. It is generally believed that miRNAs can prevent its translation or induce the degradation of target mRNA. Studies have found that miR-195 can participate in scar repair and progression, and its abnormally high expression is one of the important mechanisms for the occurrence and development of IUA [5]. Zhou et al. had found that the low expression of miR-29a may be involved in the formation and development of IUA [6]. It can be seen that miRNAs play a very important role in the treatment of IUA. In previous studies, the proportion of fibroid tissue components of the uterine muscle wall in patients with intrauterine adhesions reached 50%-80%, and uterine scar contraction or intrauterine adhesions resulted in uterine sclerosis and even anatomical structure abnormalities [7]. Excessive deposition of extracellular matrix (ECM) in place of normal endometrium is also characteristic of endometrial fibrosis [8]. Studies have shown that miR-513a-5p was downregulated in IUA and negatively regulated in ADAM-9, and miR-543 was also downregulated in IUA and negatively regulated in CDH2 and COL16A1 [7].

Current studies have shown that the key to solve uterine sclerosis and abnormal anatomical structure of uterine cavity is to clarify how to regulate and accelerate the decomposition and absorption of collagen at the level of cell growth regulation and extracellular matrix molecules. The decomposition and absorption of collagen are closely related to the expression of vimentin, *α*-SMA, and fibronectin [9]. Among the many genes regulated by miR-543, many genes are related to cicatrix and fibrosis [10–12]. However, there are no studies to confirm the specific regulation mechanism of miR-543 in IUA. In this paper, we have extended the previous study of miR-543 and explore its role and molecular mechanism in uterine adhesion through a series of experiments.

## 2. Method

### 2.1. Cell Culture and Grouping

Endometrial epithelial cells and endometrial adenocarcinoma cells were purchased from Guangzhou RiboBio Co., Ltd. (Guangzhou, China). The DMEM medium containing 10% fetal bovine serum (FBS), 100 mg/mL penicillin, and 100 U/mL streptomycin was cultured in a constant temperature incubator at 37°C and 5% CO_2_ saturated humidity. The cells were divided into four groups: miR-543 overexpression negative control group (miR-543-mimic NC), miR-543 overexpression group (miR-543 mimics), miR-543 low expression negative control group (miR-543 inhibitor NC), and miR-543 low expression group (miR-543 inhibitor). When transfected, the medium was changed to serum-free medium without penicillin and streptomycin. When the fusion degree of cell culture reached about 70%, according to the description of Lipofectamine reagent, miR-543 mimic NC, miR-543 mimics, miR-543 inhibitor NC, and miR-543 inhibitor were transfected into primary endometrial epithelial cells and endometrial adenocarcinoma cells, respectively.

### 2.2. CCK-8

After transfection, the cells in each group were cultured in good condition and digested with trypsin, and the cell density was adjusted to 5 × 10^4^ cells/mL. The 5000 cell suspension of 100 *μ*L was inhaled into the 96-well plate, and the culture continued at 37°C in the 5% CO_2_ incubator for 24 h, 48 h, 72 h, and 96 h. During the detection, 10 *μ*L of CCK-8 solution was added to the back plate and incubated in the incubator for 1-4 h. The absorbance and the ability of cell proliferation were measured at 450 nm by enzyme labeling instrument.

### 2.3. Scratch Test

The transfected and cultured cells in good condition were digested with trypsin and inoculated into a 24-well plate for overnight culture. On the next day, a vertical line was drawn in the hole with the sterile gun head perpendicular to the horizontal line at the bottom of the petri dish. PBS was rinsed twice and added serum-free medium. The cells were photographed again 24 h and 48 h later, and the cell migration was observed.

### 2.4. Transwell Invasion Assay

The matrix glue was thawed slowly on the ice and diluted with serum-free medium at 1 : 8. The Transwell cell was incubated with 200 *μ*L diluted matrix glue at 37°C for 2 h, and the residual medium was removed. The cell concentration was diluted with DMEM medium containing 10% FBS, 100 *μ*L of cell suspension was added to each Transwell chamber with matrix glue, and 600 *μ*L of DMEM medium containing 20% FBS was added under the 24-well plate and cultured in the incubator for 40 h. Then, the cells in the upper compartment were wiped with cotton swabs, fixed with methanol for 30 min, and stained with 0.1% crystal violet dye for 10 min. The membrane was removed, fixed on a slide, and randomly photographed under a microscope.

### 2.5. Flow Cytometry

After digesting each group of cells with 0.25% trypsin, the digestion was terminated with the culture medium and washed twice with PBS. Cells were collected by centrifugation and suspended by 500 *μ*L binding buffer. After adding 5 *μ*L Annexin V-FITC and 5 *μ*L propidium iodide into the mixture, the cells of each group were washed with PBS for 5-15 min, at room temperature, and the results were determined by flow cytometry.

### 2.6. RT-qPCR

Total RNA was extracted by TRIzol reagent (15596-026, Life). RNA was reverse transcribed into cDNA by ReverAid First Strand cDNA Synthesis Kit (K1622, Thermo). Then, the quantitative experiment of qPCR was carried out with GoTaq qPCR Master Mix Kit. The RT-qPCR reaction process is as follows: predenatured 10 min at 95°C, denaturation for 15 s at 95°C, and extension at 60°C for 1 min for 40 cycles. The 2^-△△CT^ method was used for relative quantitative analysis. The sequence of primers used in this experiment is shown in Table 1.

### 2.7. Western Blot

The total protein was extracted by RIPA, and the protein concentration was determined by the BCA method. Protein was separated on 10% SDS-PAGE gel and transferred to PVDF membrane. Then, the membranes were blocked with 5% nonfat at room temperature for 1 h and washed with TBST membrane for 3 times. The membranes were incubated with the primary antibody anti-CDH2 protein antibody (1RV 1000 diluted, A0432 negative), anti-CDH1 protein antibody (1RV 1000 diluted, 3195 CST), anti-Vimentin protein antibody (1RV 1000 diluted, bs-0756R, BIOSS), anti-*α*-SMA protein antibody (1RV 1000 diluted, ZB019, YTHX), and *β*-actin (1PV2000 diluted, AP0060, Bioword) overnight at 4°C. On the second day, the mice were rewarded for 30 min and washed with 10 min for 3 times, and the corresponding secondary antibodies (diluted anti-mouse HRP, 1 : 5000, item number 00001-1, ProteinTech) were added and incubated at room temperature for 3 times. Add ECL reaction for 1 min, absorb the excess photoluminescence solution, expose in darkroom, and scan the strip by scanner.

### 2.8. Luciferase Reporter Assay

The 3′-UTR region of the wild-type *CDH2* gene was cloned into luciferase vector plasmid (WT), and *CDH2* was mutated with the miR-543 binding region to obtain mutant plasmid (MUT). When 293 T cells were fused to 60%-70%, wild-type and mutant reporter plasmids of *CDH2* and miR-543 mimics were transfected into the cells. After 24 h of continuous culture, the cells were digested down, and luciferase substrates were added, respectively, to determine the fluorescence values. Luciferase activity was measured with a luciferase assay kit (E1910, Promega).

### 2.9. Statistical Analysis

All data were expressed as mean ± standard deviation (SD) and were statistically analyzed by SPSS 22.0. *t*-test was used to compare the two groups. *P* value <0.05 was considered statistically significant .

## 3. Results

### 3.1. miR-543 Inhibited the Proliferation of Endometrial Epithelial Cells and Endometrial Adenocarcinoma Cells

**Figure 1:**
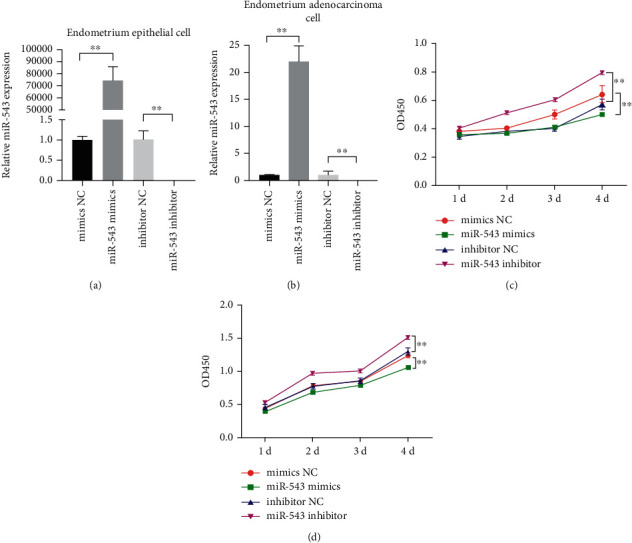
miR-543 inhibits the proliferation of endometrial epithelial cells and endometrial adenocarcinoma cells. (a) and (b) QPCR were used to detect the expression of miR-543 after transfection of miR-543 mimics and inhibitor. (c) and (d) CCK8 were used to detect the proliferation of endometrial epithelial cells and endometrial adenocarcinoma cells. The average (SD) of three independent experiments is shown in the statistical chart. ^∗∗^ indicates *P* < 0.01.

In order to detect the effect of the miR-543 expression on cell proliferation, we first transfected miR-543 mimics and miR-543 inhibitor in endometrial epithelial cells and endometrial adenocarcinoma cells. RT-qPCR results confirmed that miR-543 mimics and miR-543 inhibitor could upregulate and downregulate the expression of miR-543, respectively (Figures 1(a) and 1(b)). CCK8 was used to detect the level of cell proliferation. In primary endometrial epithelial cells (Figure 1(c)) and endometrial adenocarcinoma cells (Figure 1(d)), the overexpression of miR-543 could inhibit cell proliferation, but interfere with the expression of miR-543 and decrease the ability of cell proliferation.

### 3.2. miR-543 Promoted Apoptosis of Endometrial Epithelial Cells and Endometrial Adenocarcinoma Cells

**Figure 2:**
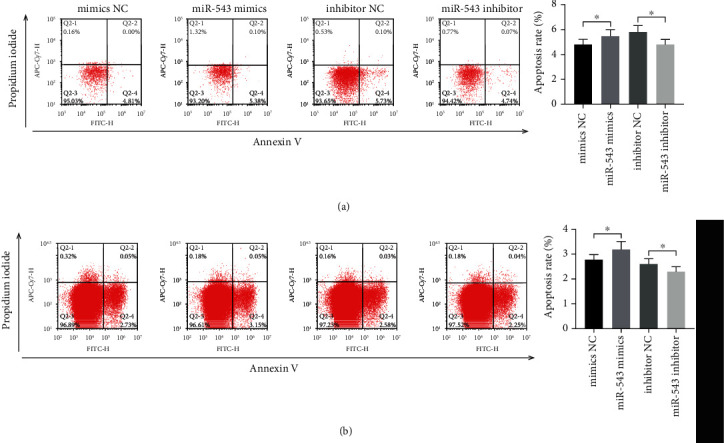
miR-543 can promote apoptosis of endometrial epithelial cells and endometrial adenocarcinoma cells. (a) The apoptosis of primary endometrial epithelial cells was detected by flow cytometry. (b) the apoptosis of endometrial adenocarcinoma cells was detected by flow cytometry. The average (SD) of three independent experiments is shown in the statistical chart, ^∗^ indicates *P* < 0.05.

The level of apoptosis induced by miR-543 was detected by flow cytometry. In primary endometrial epithelial cells (Figure 2(a)) and endometrial adenocarcinoma cells (Figure 2(b)), the apoptosis rate increased after the overexpression of miR-543 compared with the miR-543 mimic NC group. Compared with the miR-543 inhibitor NC group, the apoptosis rate of the miR-543 inhibitor group decreased slightly (*P* < 0.05).

### 3.3. miR-543 Inhibited the Migration and Invasion of Endometrial Epithelial Cells and Endometrial Adenocarcinoma Cells

**Figure 3:**
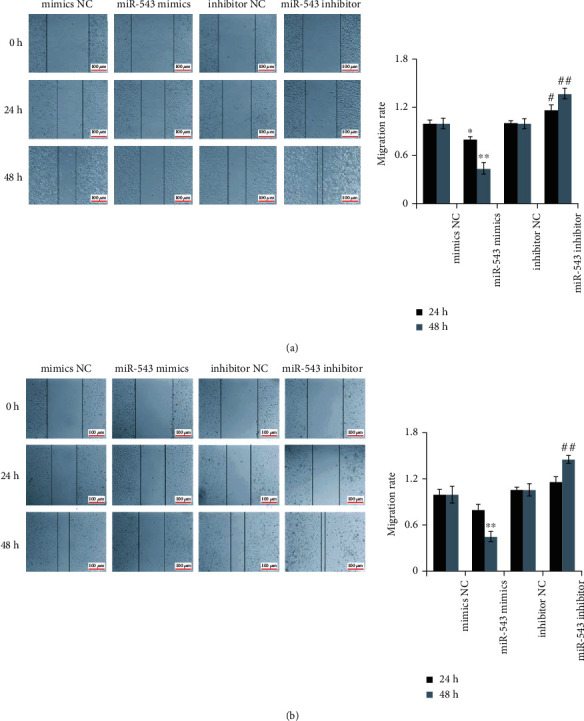
miR-543 inhibits the migration of endometrial epithelial cells and endometrial adenocarcinoma cells. (a) The results of the scratch test of primary endometrial epithelial cells. (b) The results of the scratch test of endometrial adenocarcinoma cells. The average (SD) of three independent experiments is shown in the statistical chart, ^∗^*P* < 0.05 or ^∗∗^*P* < 0.01 vs mimics NC; #*P* < 0.05 or ##*P* < 0.01 vs inhibitor NC.

The scratch test was used to detect the effect of miR-543 on cell migration. The results of the scratching test of endometrial epithelial cells (Figure 3(a)) and endometrial adenocarcinoma cells (Figure 3(b)) showed that there was no significant difference in the degree of cell migration to the center within 24 hours in the miR-543 mimic group compared with the miR-543 mimic NC group. After 48 hours, the degree of migration to the center in the miR-543 mimic group was lower than that in the miR-543 mimic NC group; that is, the ability of cell migration decreased after the overexpression of miR-543. The degree of migration to the center in the miR-543 inhibitor group was significantly higher than that in the miR-543 inhibitor NC group after 48 h. It can be seen that interfering with the expression of miR-543 can enhance the ability of cell migration.

**Figure 4:**
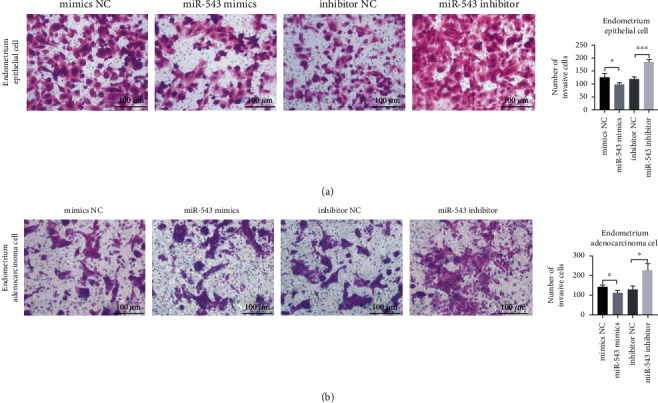
miR-543 inhibits the invasion of endometrial epithelial cells and endometrial adenocarcinoma cells. (a) Transwell was used to detect the invasive ability of endometrial epithelial cell. Magnification ×200. (b) Endometrial adenocarcinoma cell line. Magnification ×200. The average (SD) of three independent experiments is shown in the statistical chart. ^∗^ indicates *P* < 0.05; ^∗∗^ indicates *P* < 0.01.

Furthermore, we used the Transwell assay to detect the effect of miR-543 on the invasive ability of cells. In endometrial epithelial cells (Figure 4(a)) and endometrial adenocarcinoma cells (Figure 4(b)), the invasive ability of the miR-543 mimic group was significantly weaker than that of the miR-543-mimic NC group, and the invasive ability of the miR-543 inhibitor group was significantly higher than that of the miR-543 inhibitor NC group.

### 3.4. *CDH2* Is the Direct Target Gene of miR-543

**Figure 5:**
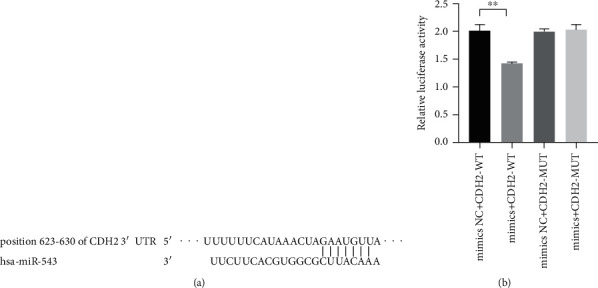
CDH2 is the target gene of miR-543. (a) The binding site of miR-543 and CDH2. (b) miR-543 inhibited luciferase reporter gene activity in THE WT group. The average (SD) of three independent experiments is shown in the statistical chart. ^∗∗^ indicates *P* < 0.01.

**Figure 6:**
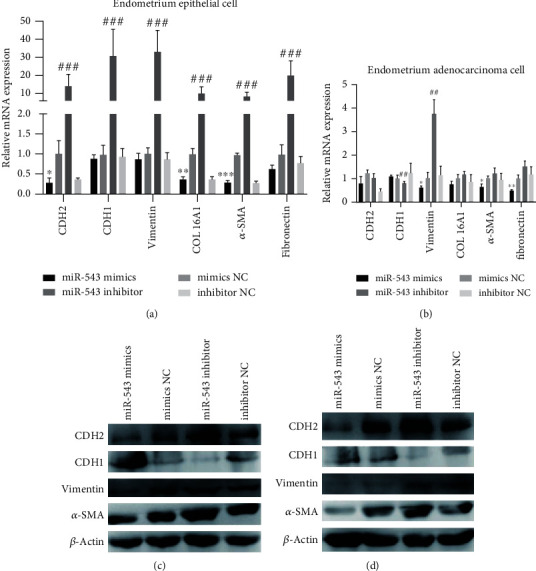
The effect of miR-543 on the expression of fibrosis-related proteins. RT-qPCR detected the expressions of CDH2, CDH1, vimentin, COL16A1, *α*-SMA, and fibronectin in endometrial epithelial cells (a) and in endometrial adenocarcinoma cells (b). WB detected the expression of CDH2, CDH1, vimentin, COL16A1, and *α*-SMA protein in endometrial epithelial cell (c) and in endometrial adenocarcinoma cell line (d). The average (SD) of three independent experiments is shown in the statistical chart. ^∗^*P* < 0.05 or ^∗∗^*P* < 0.01 vs mimics NC; #*P* < 0.05 or ##*P* < 0.01or ###*P* < 0.001 vs inhibitor NC.

We predicted that *CDH2* is a potential target gene of miR-543 through TargetScan software (Figure 5(a)). The results of the double luciferase reporter gene detection (Figure 5(b)) showed that the overexpression of miR-543 significantly inhibited the activity of the wild-type *CDH2* 3′-UTR luciferase reporter gene, but there was no significant difference between the two groups in *CDH2*-MUT. This result indicates that *CDH2* is the target gene of miR-543. We observed that the mRNA and protein levels of CDH2 decreased significantly after the miR-543 overexpression (Figure 6). And it was further confirmed that miR-543 was directly targeted to *CDH2*.

### 3.5. miR-543 Regulates the Expression of Fibrosis-Related Genes in Cells

In order to further clarify the mechanism of miR-543 regulating the biological function of cells, we detected the candidate genes responsible for regulating fibrosis in endometrial cells and endometrial adenocarcinoma cells transfected with miR-543 mimics or inhibitor by RT-qPCR and WB. In endometrial epithelial cells (Figure 6(a)) and endometrial adenocarcinoma cells (Figure 6(b)), after the overexpression of miR-543, the mRNA expression of *vimentin, COL16A1, α-SMA*, and *Fibronectin* decreased, while the mRNA expression of *CDH1* increased, while miR-543 inhibitor played the opposite role.

The results of Figures 6(c) and 6(d) showed that the protein expression of CDH2, vimentin, and *α*-SMA in the miR-543 mimic group was lower than that in the mimic NC group, while the protein expression of CDH1 was increased in the miR-543 mimic group. Compared with inhibitor NC group, the protein expression of CDH2, Vimentin and *α*-SMA in the miR-543 inhibitor group increased, while the protein expression of CDH1 decreased. These results confirmed that miR-543 can inhibit the expression of cell fibrosis protein and thus inhibit uterine adhesion.

## 4. Discussion

IUA refers to the injury of the basal layer of endometrium caused by pregnant or nonpregnant uterine trauma and even complete occlusion of the uterine cavity or cervix, such as secondary amenorrhea, periodic abdominal pain, recurrent abortion, and infertility [13, 14]. Previous studies have confirmed that the expression of miR-543 is downregulated in IUA and negatively regulates *CDH2* [7]. As a one-way transmembrane glycoprotein, *CDH2* is associated with ECM because it plays a certain role in maintaining cell-to-cell adhesion [7]. In this paper, an experiment was designed to explore the influence and mechanism of miR-543, *CDH2*, and related genes in IUA.

After grouping culture, the migration ability, proliferation level, apoptosis level, migration level, and invasion ability of endometrial epithelial cells and cancer cells were detected by scratch test, CCK-8 detection, flow cytometry, and Transwell test. The results showed that after the overexpression of miR-543, the migration ability of endometrial epithelial cells and endometrial adenocarcinoma cells decreased, the proliferation rate slowed down, the invasive ability also decreased, and the apoptosis rate increased. It can be seen that the upregulation of miR-543 level can reduce cell viability and cell function. After interfering with the expression of miR-543, the cell function was opposite to that mentioned above. These results suggest that upregulation of miR-543 can slow down the proliferation of endometrial epithelial cells and endometrial adenocarcinoma cells.

*CDH1* is considered to be a tumor suppressor gene and a tumor metastasis suppressor gene. As a member of the cadherin family, *CDH2* exists in the nervous system [15] and is also involved in the occurrence and metastasis of tumor cells. CDH2 is called mesenchymal-specific cadherin [16, 17] and is a key regulator of mesenchymal stem cell differentiation and fate [5, 6, 18]. During skin wound healing, primary fibroblasts changed the expression of cadherin in *α*-SMA-positive myofibroblasts from *CDH2* to *CDH11*. The differentiation of myofibroblasts is related to the increase of CDH11 and the decrease of CDH2 level as well as the functional phenotypic changes of pathogenic fibroblasts. Neurons mainly express CDH2, while epithelial cells highly express E-cadherin (*CDH1*). When cells undergo epithelial-mesenchymal transformation of (EMT), *CDH1* is downregulated while *CDH2* is upregulated. EMT marker E-cadherin decreased, while N-cadherin increased. In this study, the results showed that the expression of miR-543 was negatively correlated with the expression of *CDH2* and positively correlated with the expression of *CDH1* in endometrial epithelial cells and endometrial adenocarcinoma cells. In addition, the results of the double luciferase assay showed that miR-543 could directly bind to *CDH2*, indicating that the high expression of miR-543 may inhibit the endothelial interstitial transformation of endometrial epithelial cells by inhibiting the expression of EMT protein *CDH2* and upregulating the expression of *CDH1*.

*Vimentin* is also considered as a specific marker of epithelial-mesenchymal transformation, which is closely related to tumor growth and metastasis [19]. *α*-SMA is a marker protein of myofibroblasts. In some pathological conditions, epithelial cells can transdifferentiate into myofibroblasts and express *α*-SMA. The expression level of *α*-myofibroblasts can indirectly reflect the degree of epithelial pathological changes. Some studies have shown that vimentin and *α*-SMA are highly expressed when renal tubular epithelial cells differentiate into interstitial cells and have the characteristics of fibrous cells [20]. Fibronectin plays a very important role in wound repair and healing and is a key substance to promote wound healing [9]. In this study, it was confirmed that the expression of miR-543 was upregulated, while the expression of *α*-SMA, vimentin, and fibronectin was decreased in endometrial epithelial cells and endometrial adenocarcinoma cells. When the expression of miR-543 was inhibited, the expression of *α*-SMA, vimentin, and fibronectin in primary endometrial epithelial cells and endometrial adenocarcinoma cells increased. These results further confirmed that miR-543 can inhibit the expression of fibroproteins and inhibit uterine adhesion.

Then, we studied the regulation of the collagen COL16A1 expression by miR-543. The results of RT-qPCR detection showed that the expression of miR-543 was upregulated, and the mRNA expression of *COL16A1* was decreased in primary endometrial epithelial cells and endometrial adenocarcinoma cells. Grässel et al. [21] reported that COL16A1 is secreted and synthesized by uterine stromal cells, which can affect the decomposition and absorption of collagen. The decomposition and absorption of collagen are the key to scar healing. Some studies have shown that when intestinal inflammation occurs and intestinal tissue fibrosis occurs, the secretion of COL16 increases [22, 23]. Upregulation of miR-543 can inhibit the expression of COL16A1, which may degrade collagen deposition.

The experimental results showed that when miR-543 was low expressed in endometrial epithelial cells, the expression of CDH2 was increased, the expression of CDH1 was downregulated, and fibroproteins such as vimentin, *α*-SMA, and fibronectin were highly expressed, which showed severe endometrial fibrosis. The decrease of the COL16A1 expression after upregulation of miR-543 also indicated that the inhibition of collagen decomposition and absorption in the tissue also made the scar unable to recover. According to this experiment, it is inferred that during the formation of uterine adhesion, miR-543 can specifically inhibit the expression of CDH2 and further inhibit the expression of vimentin, *α*-SMA, fibronectin, and COL16A1, thus inhibit the degree of fibrosis and collagen content in uterine adhesion tissue and participate in the occurrence and development of uterine adhesion.

## Figures and Tables

**Table 1:** Primer sequence.

Gene	Primer sequence (5′ − 3′)
CDH1	F: ATTTTTCCCTCGACACCCGAT
	R: TCCCAGGCGTAGACCAAGA
CDH2	F: AGCCAACCTTAACTGAGGAGT
	R: GGCAAGTTGATTGGAGGGATG
COL16A1	F: CCACCAGAAGACGTGGTATCT
	R: CAGGACACAAAGTCGCCATC
Vimentin	F: GACGCCATCAACACCGAGTT
	R: CTTTGTCGTTGGTTAGCTGGT
*α*-SMA	F: GTGTTGCCCCTGAAGAGCAT
	R: GCTGGGACATTGAAAGTCTCA
Fibronectin	F: AGGAAGCCGAGGTTTTAACTG
	R: AGGACGCTCATAAGTGTCACC
hsa-miR-543	F: AAACATTCGCGGTGCACTTCTT
GAPDH	F: CCAGAAGACTGTGGATGGCC
	R: CATGCCAGTGAGCTTCCC
U6	F: CTCGCTTCGGCAGCACA
	R: AACGCTTCACGAATTTGCGT

## Data Availability

Data are available in the article.

## References

[1] Salma U., Xue M., Md Sayed A. S., Xu D. (2014). Efficacy of intrauterine device in the treatment of intrauterine adhesions. *BioMed research international*.

[2] Yan Y., Xu D. (2018). The effect of adjuvant treatment to prevent and treat intrauterine adhesions: a network meta-analysis of randomized controlled trials. *Journal of Minimally Invasive Gynecology*.

[3] Hooker A. B., Lemmers M., Thurkow A. L. (2014). Systematic review and meta-analysis of intrauterine adhesions after miscarriage: prevalence, risk factors and long-term reproductive outcome. *Human Reproduction Update*.

[4] Tam W. H., Lau W. C., Cheung L. P., Yuen P. M., Chung T. K. (2002). Intrauterine adhesions after conservative and surgical management of spontaneous abortion. *The Journal of the American Association of Gynecologic Laparoscopists*.

[5] LG W. J. J., Zhang Y. D. (2020). Expression of miR-195 in intrauterine adhesions and its relationship with TGF-*β*1/Smads,FGF2/FHFR1/ERK pathways. *Journal of Hainan Medical University*.

[6] Zhou M., He Y., Liu F. (2014). Expression and significance of miR-29a, TGF-*β*1, Smad2 and Smad3 in endometrium of patient with intrauterine adhesions. *Journal of Practical Medicine*.

[7] Liu X., Duan H., Zhang H. H., Gan L., Xu Q. (2016). Integrated data set of microRNAs and mRNAs involved in severe intrauterine adhesion. *Reproductive Sciences*.

[8] Diegelmann R. F., Evans M. C. (2004). Wound healing: an overview of acute, fibrotic and delayed healing. *Frontiers in Bioscience*.

[9] Maquart F. X., Monboisse J. C. (2014). Matrice extracellulaire et cicatrisation. *Pathologie Biologie*.

[10] Fuentes-Mattei E., Bayraktar R., Manshouri T. (2020). miR-543 regulates the epigenetic landscape of myelofibrosis by targeting TET1 and TET2. *JCI Insight*.

[11] Ouyang F., Liu X., Liu G. (2020). Correction for: Long non-coding RNA RNF7 promotes the cardiac fibrosis in rat model via miR-543/THBS1 axis and TGF*β*1 activation. *Aging (Albany NY)*.

[12] Zhu H. Y., Bai W. D., Wang H. T. (2016). Peroxisome proliferator-activated receptor-*γ* agonist inhibits collagen synthesis in human keloid fibroblasts by suppression of early growth response-1 expression through upregulation of miR-543 expression. *American Journal of Cancer Research*.

[13] Ishibashi N., Maebayashi T., Asai-Sato M., Kawana K., Okada M. (2019). Radiation therapy for vaginal cancer in complete uterine prolapse with intrauterine adhesion: a case report. *BMC Womens Health*.

[14] Liu Z., Kong Y., Gao Y. (2019). Revealing the interaction between intrauterine adhesion and vaginal microbiota using high-throughput sequencing. *Molecular Medicine Reports*.

[15] Luense L. J., Veiga-Lopez A., Padmanabhan V., Christenson L. K. (2011). Developmental programming: gestational testosterone treatment alters fetal ovarian gene expression. *Endocrinology*.

[16] Agarwal S. K., Lee D. M., Kiener H. P., Brenner M. B. (2008). Coexpression of two mesenchymal cadherins, cadherin 11 and N-cadherin, on murine fibroblast-like synoviocytes. *Arthritis and Rheumatism*.

[17] Chang S. K., Noss E. H., Chen M. (2011). Cadherin-11 regulates fibroblast inflammation. *Proceedings of the National Academy of Sciences of the United States of America*.

[18] Alimperti S., Andreadis S. T. (2015). CDH2 and CDH11 act as regulators of stem cell fate decisions. *Stem Cell Research*.

[19] Satelli A., Li S. (2011). Vimentin in cancer and its potential as a molecular target for cancer therapy. *Cellular and Molecular Life Sciences*.

[20] Lin J. J., Bai M., Zhuang Y. B. (2016). The role of PEA3 in albumin induced injury of renal tubular epithelial cells. *Journal of Nanjing Medical University*.

[21] Grässel S., Bauer R. J. (2013). Collagen XVI in health and disease. *Matrix Biology*.

[22] Ratzinger S., Eble J. A., Pasoldt A. (2010). Collagen XVI induces formation of focal contacts on intestinal myofibroblasts isolated from the normal and inflamed intestinal tract. *Matrix Biology*.

[23] Li C., Nguyen H. T., Zhuang Y. (2011). Post-transcriptional up-regulation of miR-21 by type I collagen. *Molecular Carcinogenesis*.

